# European Psychiatry: 2024 in review

**DOI:** 10.1192/j.eurpsy.2025.4

**Published:** 2025-01-27

**Authors:** Sophia Frangou, Andrea Fiorillo

**Affiliations:** 1 Icahn School of Medicine at Mount Sinai, New York, NY, USA; 2 Department of Psychiatry, University of British Columbia, Vancouver, Canada; 3 University of Campania “Luigi Vanvitelli”, Italy

As we reflect on 2024, *European Psychiatry* continues to grow in its influence, showcasing exceptional research that advances our understanding of mental health and psychiatric care. Our editorial team has worked tirelessly to uphold the highest ethical and publishing standards and to present groundbreaking research to our readers.

This past year, we featured several key studies, including topics such as youth mental health [1], addictions [2], mood disorders [3–5], suicide [6], and fetal and postpartum mental health [7-9]. We also explored the therapeutic potential and challenges of emerging treatments, including psychedelics [10–11] and neuromodulation [12].

Submissions to *European Psychiatry* continue to rise, reflecting the journal’s reputation as a trusted outlet for high-impact research. In 2024, we received 627 submissions and published 88 papers following rigorous peer review, ensuring that only the most innovative and informative studies were accepted. Other metrics establishing the influence of *European Psychiatry* in 2024 include the Journal Impact Factor, which is 7.2, and the more than 1.5 million downloads of articles from our website. Finally, *European Psychiatry* is ranked 15th among 279 psychiatry journals based on Clarivate Analytics. Table 1 showcases our top five articles published in 2024 with the highest number of views/downloads from the Cambridge Core platform, the top five articles based on Altmetric scores reflecting their impact in the news and social media [13], and the top five articles with the highest number of citations based on data from Dimensions.ai [14]. This success is due, in no small part, to the dedication of our expert peer reviewers and our editorial team, whose contributions are instrumental in maintaining the journal’s excellence.

**Table 1.** Most influential papers published in 2024

Most downloaded papers	N of downloads
Effects of psychedelic microdosing versus conventional ADHD medication use on emotion regulation, empathy, and ADHD symptoms in adults with severe ADHD symptoms: A naturalistic prospective comparison study [11]	24139
Prevalence of attention-deficit hyperactivity disorder (ADHD): systematic review and meta-analysis [18]	3923
A systematic review and meta-analysis of music interventions to improve sleep in adults with mental health problems [19]	2396
Efficacy and tolerability of FDA-approved atypical antipsychotics for the treatment of bipolar depression: a systematic review and network meta-analysis [20]	2336
Mortality in patients with major depressive disorder: A nationwide population-based cohort study with 11-year follow-up [4]	2079

Most attention in media and social media	Altmetric Score
Prenatal maternal PTSD as a risk factor for offspring ADHD: A register-based Swedish cohort study of 553 766 children and their mothers [21]	50
Baseline symptom severity and efficacy of Silexan in patients with anxiety disorders: A symptom-based, patient-level analysis of randomized, placebo-controlled trials [22]	30
Effects of psychedelic microdosing versus conventional ADHD medication use on emotion regulation, empathy, and ADHD symptoms in adults with severe ADHD symptoms: A naturalistic prospective comparison study [11]	24
Clinician treatment choices for post-traumatic stress disorder: ambassadors survey of psychiatrists in 39 European countries [23]	14
Long-term benzodiazepine use and risk of labor market marginalization in Finland: A cohort study with 5-year follow-up [24]	14

Most cited	N of citations
Climate change and mental health: Position paper of the European Psychiatric Association [16]	6
The role of cognitive reserve and clinical symptoms in the association between genetic liability for educational attainment and functioning in first-episode psychosis: a mediation analysis. [25]	5
Mental health in Ukraine in 2023 [26]	4
Efficacy and tolerability of FDA-approved atypical antipsychotics for the treatment of bipolar depression: a systematic review and network meta-analysis [20]	4
Baseline symptom severity and efficacy of Silexan in patients with anxiety disorders: A symptom-based, patient-level analysis of randomized, placebo-controlled trials [22]	3

Downloads from Cambridge Core (https://www.cambridge.org/core/journals/european-psychiatry/); Altmetric Scores based on https://www.altmetric.com/; Citations based on https://app.dimensions.ai/. Data correct as of January 6th 2025.

The scope of the journal extends beyond research articles and includes Viewpoints, Guidance, and Position papers. Viewpoints provide concise, expert opinions or perspectives on current issues, emerging trends, or challenges in psychiatry and mental health. These articles aim to stimulate discussion, propose innovative ideas, or offer critical analyses of policies, practices, or research findings, often addressing topics at the intersection of science, clinical practice, and societal impact. They are typically evidence-informed but focus on interpretation and thought leadership rather than presenting original research. As *European Psychiatry* is the voice of the European Psychiatric Association, we publish Position and Guidance papers that articulate the official stance or perspective of the association on specific topics in psychiatry or mental health. Position papers are grounded in evidence and provide a comprehensive analysis of a topic, outlining key challenges, supporting data, and proposed recommendations. These papers aim to influence policy or shape research priorities, offering a clear and authoritative voice on critical issues within the field. In 2024, our position papers explored the importance of digitization for mental health [15] and the mental health challenges of climate change [16].

Our Guidance papers provide practical recommendations or frameworks to inform clinical practice or policy development in psychiatry and mental health. These papers focus on translating current knowledge into actionable steps, addressing gaps in practice, or standardizing approaches within the field. They aim to assist clinicians, researchers, and policymakers in making informed decisions and improving outcomes. In 2024, our Guidance paper provided a synthesis of the evidence on lifestyle interventions for adults with severe mental illness [17]. Finally, our editorial team is also active during the annual meetings of the association where workshops on scientific writing and publishing are organized.

We continue to focus on diversity, and inclusion, ensuring that the journal reflects broad perspectives while also highlighting success and challenges within Europe. We note our ongoing open call for articles on the theme of “Population Neuroscience Perspectives of Psychopathology.” This special collection aims to showcase how large-scale studies can enhance our understanding of the underpinnings of mental disorders. Contributions with data from diverse populations are particularly encouraged

Looking ahead, we aim to expand our content further. We are pleased to announce a special call for papers on the transformative role of artificial intelligence (AI) in psychiatry and mental health. We invite submissions that explore the application of AI across all areas of psychiatry and mental health, including but not limited to the following: (a) AI-based diagnostic tools and predictive models; (b) Machine learning applications in treatment personalization and response prediction; (c) Ethical challenges and considerations in deploying AI in mental health care; (d) AI-driven advancements in neuroimaging and computational psychiatry; (e) The role of AI in addressing mental health disparities and improving access to care; and (f) Interdisciplinary approaches combining AI with other emerging technologies. Submissions may include original research, reviews and viewpoints that critically assess the potential as well as the limitations, and future directions of AI in psychiatry.

As we enter 2025, we thank our authors, reviewers, and readers for their continued support. Together, we will continue to advance the field where *European Psychiatry* serves as a platform for meaningful and transformative research.

## References

[1] Iorfino F, Oliveira R, Cripps S, et al. A prognostic model for predicting functional impairment in youth mental health services. Eur Psychiatry 2024;67(1):e87. 39697104 10.1192/j.eurpsy.2024.1787 PMC11733617

[2] Billaux P, Segobin S, Maillard A, et al. Let’s focus on the insula in addiction: a refined anatomical exploration of insula in severe alcohol and cocaine use disorders. Eur Psychiatry 2024;67(1):e75. 39543913 10.1192/j.eurpsy.2024.1784 PMC11730057

[3] Roux P, Frileux S, Vidal N, et al. Relationships between cognition, functioning, and quality of life of euthymic patients with bipolar disorder: structural equation modeling with the FACE-BD cohort. Eur Psychiatry 2024;67(1):e78. 39543921 10.1192/j.eurpsy.2024.1789 PMC11730061

[4] Bitter I, Szekeres G, Cai Q, et al. Mortality in patients with major depressive disorder: A nationwide population-based cohort study with 11-year follow-up. Eur Psychiatry 2024;67(1):e63. 39344202 10.1192/j.eurpsy.2024.1771 PMC11536202

[5] Borroni E, Buoli M, Nosari G, et al. Impact of air pollution exposure on the severity of major depressive disorder: results from the DeprAir study. Eur Psychiatry 2024;67(1):e61. 39328146 10.1192/j.eurpsy.2024.1767 PMC11457114

[6] Jollant F, Leon C. Suicidal transition rates and their predictors in the adult general population: a repeated survey over 21 years in France. Eur Psychiatry 2024;67(1):e74. 39468715 10.1192/j.eurpsy.2024.1782 PMC11730064

[7] Doncarli A, Demiguel V, Le Ray C, et al. Prevalence of anxiety symptoms and associated factors at 2 months postpartum, results from a 2021 French national prospective cohort study. Eur Psychiatry 2024;67(1):e89. 39726376 10.1192/j.eurpsy.2024.1799 PMC11733619

[8] Chan JKN, Lee KCK, Wong CSM, Chang WC. Risk of congenital malformations associated with first-trimester exposure to antipsychotics: a propensity score-weighted population-based cohort study. Eur Psychiatry 2024;67(1):e42. 38800849 10.1192/j.eurpsy.2024.1758 PMC11441336

[9] Tebeka S, Gloaguen E, Mullaert J, et al. Genome-wide association study of early-onset and late-onset postpartum depression: the IGEDEPP prospective study. Eur Psychiatry 2024;67(1):e35. 38555957 10.1192/j.eurpsy.2024.26 PMC11059250

[10] Liu H, Wang C, Lan X, et al. Functional connectivity of the amygdala subregions and the antidepressant effects of repeated ketamine infusions in major depressive disorder. Eur Psychiatry 2024;67(1):e33. 38572583 10.1192/j.eurpsy.2024.1744 PMC11059247

[11] Haijen ECHM, Hurks PPM, Kuypers KPC. Effects of psychedelic microdosing versus conventional ADHD medication use on emotion regulation, empathy, and ADHD symptoms in adults with severe ADHD symptoms: A naturalistic prospective comparison study. Eur Psychiatry 2024;67(1):e18. 38351594 10.1192/j.eurpsy.2024.8 PMC10966614

[12] Wu GR, Baeken C. Exploring potential working mechanisms of accelerated HF-rTMS in refractory major depression with a focus on locus coeruleus connectivity. Eur Psychiatry 2024;67(1):e70. 39417327 10.1192/j.eurpsy.2024.1769 PMC11730058

[13] Altmetric. Database. available from: https://www.altmetric.com/explorer. [accessed 6.1.25].

[14] Digital Science. Dimensions [Software]. available from: https://app.dimensions.ai. 2018; [accessed 6.1.25]. under licence agreement.

[15] Kalman JL, Burkhardt G, Samochowiec J, et al. Digitalising mental health care: practical recommendations from the European Psychiatric Association. Eur Psychiatry 2024;67(1):e4. 10.1192/j.eurpsy.2023.2466 PMC10790232 38086744

[16] Brandt L, Adorjan K, Catthoor K, et al. Climate change and mental health: position paper of the European Psychiatric Association. Eur Psychiatry 2024;67(1):e41. 38778031 10.1192/j.eurpsy.2024.1754 PMC11441337

[17] Maurus I, Wagner S, Spaeth J, et al. EPA guidance on lifestyle interventions for adults with severe mental illness: a meta-review of the evidence. Eur Psychiatry 2024; 67(1):e80. 39655999 10.1192/j.eurpsy.2024.1766 PMC11733621

[18] Popit S, Serod K, Locatelli I, Stuhec M. Prevalence of attention-deficit hyperactivity disorder (ADHD): systematic review and meta-analysis. Eur Psychiatry 2024;67(1):e68. 39381949 10.1192/j.eurpsy.2024.1786 PMC11536208

[19] Zhao N, Lund HN, Jespersen KV. A systematic review and meta-analysis of music interventions to improve sleep in adults with mental health problems. Eur Psychiatry 2024;67(1):e62. 39373544 10.1192/j.eurpsy.2024.1773 PMC11536203

[20] Li S, Xu C, Hu S, Lai J. Efficacy and tolerability of FDA-approved atypical antipsychotics for the treatment of bipolar depression: a systematic review and network meta-analysis. Eur Psychiatry 2024;67(1):e29. 38487836 10.1192/j.eurpsy.2024.25 PMC10988162

[21] Borgert M, Melin A, Hollander A-C, Rahman S. Prenatal maternal PTSD as a risk factor for offspring ADHD: a register-based Swedish cohort study of 553 766 children and their mothers. Eur Psychiatry 2024;67(1):e22. 38425211 10.1192/j.eurpsy.2024.21 PMC10966610

[22] Dold M, Möller HJ, Volz HP, et al. Baseline symptom severity and efficacy of Silexan in patients with anxiety disorders: a symptom-based, patient-level analysis of randomized, placebo-controlled trials. Eur Psychiatry 2024;67(1):e23. 38425206 10.1192/j.eurpsy.2024.16 PMC10966615

[23] Rojnic Kuzman M, Padberg F, Amann BL, et al. Clinician treatment choices for post-traumatic stress disorder: ambassadors survey of psychiatrists in 39 European countries. Eur Psychiatry 2024;67(1):e24. 38450651 10.1192/j.eurpsy.2024.19 PMC10988156

[24] Taipale H, Tanskanen A, Kurko T, et al. Long-term benzodiazepine use and risk of labor market marginalization in Finland: A cohort study with 5-year follow-up. Eur Psychiatry 2024;67(1):e34. 38572545 10.1192/j.eurpsy.2024.1745 PMC11059246

[25] Clougher D, Segura AG Forte MF, et al. The role of cognitive reserve and clinical symptoms in the association between genetic liability for educational attainment and functioning in first-episode psychosis: a mediation analysis. Eur Psychiatry 2024;1–31. 38178712 10.1192/j.eurpsy.2023.2480 PMC11795430

[26] Martsenkovskyi D, Shevlin M, Ben-Ezra M, et al. Mental health in Ukraine in 2023. Eur Psychiatry 2024;67(1):e27. 38533632 10.1192/j.eurpsy.2024.12 PMC10988158

